# Influence of Semi-Random and Regular Shot Peening on Selected Surface Layer Properties of Aluminum Alloy

**DOI:** 10.3390/ma14247620

**Published:** 2021-12-10

**Authors:** Jakub Matuszak, Kazimierz Zaleski, Agnieszka Skoczylas, Krzysztof Ciecieląg, Krzysztof Kęcik

**Affiliations:** 1 Department of Production Engineering, Mechanical Engineering Faculty, Lublin University of Technology, 20-618 Lublin, Poland; j.matuszak@pollub.pl (J.M.); k.zaleski@pollub.pl (K.Z.); k.ciecielag@pollub.pl (K.C.); 2 Department of Applied Mechanics, Mechanical Engineering Faculty, Lublin University of Technology, 20-618 Lublin, Poland; k.kecik@pollub.pl

**Keywords:** regular shot peening, semi-random shot peening, surface layer, surface roughness, microhardness, residual stress

## Abstract

This paper attempts to compare regular shot peening (RSP) and semi-random shot peening (SRSP). A characteristic of the first method is that the peening elements hit the treated surface in sequence, with a regular distance maintained between the dimples. The other method (SRSP) is a controlled modification of the shot-peening process, which is random by nature. The shot-peening method used in this study differs from conventional shot peening (shot blasting and vibratory shot peening) in that it allows controlled and repeatable determination of the configuration and distribution of impacts exerted by the peening element on the workpiece surface, which makes the process more repeatable and easier to model. Specimens of EN-AW 7075 aluminum alloy were used for testing. The following variables were used in the experiments: ball diameter, impact energy, and distance between the dimples. Microhardness distribution in the surface layer, 2D surface roughness, and surface topography were analyzed. FEM simulations of the residual stress distribution in the surface layer were performed. It has been found that regular shot peening results in reduced surface roughness, while semi-random shot peening leads to higher surface layer hardening.

## 1. Introduction

Machine components used in many industries often require adequate preparation of both surface and surface layers in order to improve surface roughness and performance properties such as fatigue strength, corrosion resistance, and wear resistance. One way to achieve this is to use mechanical surface machining, which includes processes such as shot peening and burnishing [1].

**Figure 1:**
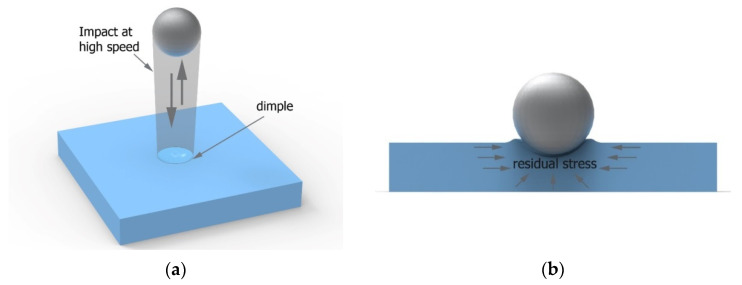
Schematic idea of the shot-peening process (**a**) and residual stress visualization (**b**).

The idea of shot peening is shown in Figure 1a. The peening element with a defined or—in the case of vibratory shot peening and blasting—undefined energy hits the workpiece and thus mirrors its shape on the machined surface. Plastic deformation is caused by a system of forces inducing surface pressures, the values of which exceed the yield stress of the machined material. The mirroring of the shape of the shot-peening element on the workpiece surface changes the arrangement of surface micro-irregularities. At the same time, work hardening, which occurs during the process, leads to changes in the properties of the surface layer, e.g., residual stresses (Figure 1b).

In the burnishing process, a hard and smooth burnishing element either hits or exerts pressure on the surface of the workpiece. Although burnishing elements usually have the shape of a sphere, cylinder, or cone, they may also be solids composed of a cylinder and a cone or of a sphere segment and a torus segment. The burnishing process is associated with plastic deformation of the workpiece surface layer, as well as with changes in the geometric structure of the workpiece surface.

The above-described methods have their specific areas of application in many industries and are the object of studies conducted by numerous research centers.

The shot-peening process is usually used to ensure specific effects such as surface layer hardening and the induction of favorable compressive residual stresses to improve fatigue strength, welded joint strength [2], and wear and corrosion resistance. Another effect is to reduce surface roughness or to create a specific system of surface micro-irregularities in order to achieve the required functional properties, e.g., the formation of lubricating micro-grooves on the mating surfaces under frictional conditions, e.g., in journals, bushings, guides, cylinders, pistons, etc. Brushing is an unconventional machining method that produces effects that are similar to those obtained with the shot-peening process. This method combines the features of both machining (loss of processed material) and shot peening (strengthened surface layer, increased microhardness, and induction of desirable compressive residual stresses) [3,4,5].

The main effects of shot peening include improved fatigue strength due to the creation of compressive residual stresses and surface layer hardening. In addition to the distribution of residual stresses and hardness, the factors affecting fatigue strength include the mechanical properties of a material and the formation of a specific system of surface micro-irregularities resulting from the mirroring of the shot-peening element on the surface of the workpiece [6]. The effect of shot peening on the fatigue strength of aluminum alloys was investigated in [7,8,9,10,11]. The authors of [7] compared the fatigue strength of 7050-T7451 alloy after polishing, machining, and shot peening. Four different combinations of media and shot-peening intensity were studied. In compliance with the ASTM E466-07 standard, cylindrical specimens were used for testing. Fatigue tests were conducted under high- and low-cycle conditions. In addition, fatigue life dispersion was analyzed. It was shown that there was a close relationship between shot size, surface roughness, and fatigue life. The same authors analyzed the fatigue life of as-polished and shot-peened specimens using a monotonic and cyclic damage model [8]. Predictions of the cyclic damage model were globally closer to experimental fatigue lives than those obtained with the monotonic damage model. In [9,10], the effect of shot-peening processing parameters on the fatigue behavior and fatigue crack propagation of aluminum AA7475-T7351 alloy was investigated. Surface roughness was found to be as important in influencing fatigue strength as residual stress. The authors of [11] investigated the effect of severe shot peening with different intensities on the fatigue life of AW 7075 aluminum alloy. Compared to mechanically polished specimens, the fatigue life of specimens after severe shot-peening treatment increased by 9% depending on the applied conditions. A comparison of the effects of vibratory peening and shot peening is presented in [12]. For Ti6Al4V titanium alloy, similar compressive stress values were obtained with shot peening and vibratory peening. For E-16NiCrMo13 steel, however, the maximum compressive residual stresses were higher and deeper after vibratory peening. The method named by the author as random controlled shot peening ensures the control of dimples by means of precisely defined movements of the peening elements, which enables the determination of impact density. The application of this method makes it possible to obtain similar increases in fatigue life as those obtained with the vibratory shot-peening process [13].

The impact of the peening element on the machined surface results in a changed pattern of surface micro-irregularities and, consequently, in changed roughness parameters. Surface roughness after shot peening primarily depends on the processing conditions, process intensity, shape and size of peening elements, shot-peening method, or properties of the machined material. Adequately selected shot-peening process conditions make it possible to significantly reduce roughness parameters or to optimize the Abbott–Firestone curve providing information about the wear rate of mating elements [12,14,15,16,17]. However, when the force exerted by the peening element is too high, the process can cause high plastic strains and thus increased roughness [18,19]. A combination of conventional shot peening and ultrasonic shot-peening machining can lead to improved surface roughness compared to the use of the shot-peening process only [19]. In terms of reducing roughness parameters, very good effects can be obtained with the use of the ball-burnishing process. In [20], the effects of burnishing speed, feed, and clamping force in ball burnishing of AISI 1045 steel were investigated. It was shown that there was a pressure burnishing force limit, beyond which an increase in surface roughness was observed. Moreover, it was found that burnishing speed only had a slight effect on surface roughness, which—in terms of efficiency—can be regarded as a premise for the use of the maximum possible values (taking into account machine tool kinematics). On the other hand, higher burnishing feed leads to increased surface roughness, with the roughness parameters being strongly dependent on the initial roughness. The stereometric state of a surface is strongly correlated with fatigue strength due to the propagation of cracks that depend on surface development. In [21], the authors studied the effect of laser peening and shot peening on surface roughness after friction stir welding. Surface properties were analyzed for both the base material and weld nugget. In the range of processing parameters applied, the highest roughness values were obtained after shot peening, while the surface roughness changes in the weld-nugget region were small for the laser- and shot-peened as well as unpeened specimens. A great advantage of shot-peening and burnishing processes is that they make it possible to process and improve the surface roughness of materials after heat treatment, as demonstrated in [22,23]. As a result of surface burnishing after turning, hardened shafts show a significant reduction in their surface roughness parameters [22]. The use of surface treatment methods such as vibratory shot peening and anodizing with vibratory shot peening makes it possible to increase the strength of titanium alloy adhesive joints [24].

Defects, damage, and burrs on the edges of elements may be corrosion centers. By surface smoothing, work hardening, and thus the induction of compressive residual stresses, shot peening may contribute to inhibiting the development of corrosion [25,26]. Aluminum alloys exposed to pitting corrosion show a drastic decrease in their fatigue strength [27]. One way to improve the material’s resistance to corrosion fatigue is by using shot peening. Shot peening can also be combined with other processes for improving corrosion resistance, e.g., plasma electrolytic oxidation (PEO) [28].

Many research works undertake finite element method (FEM) analyses of the distribution of residual stresses remaining in the surface layer after shot peening [29,30,31,32,33]. Obtained FEM results are very often highly consistent with experimental findings [34,35]. The accuracy of shot-peening process modeling is affected by, among other things, the ratio of side lengths of the finite elements used to discretize the object, the refinement of the mesh, and the finite shape of the element [36]. To represent the behavior of a material under dynamic conditions, the Johnson–Cook model is generally used for tested materials. The constitutive J-C model reflects the behavior of many materials under dynamic loads well. Despite its relatively simple form, the model considers the influence of the degree of deformation, strain rate, and temperature on the flow stress behavior. In FEM simulations of the vibratory shot-peening process, much attention is paid to correct description and modeling of the random shot-peening process wherein multiple peening elements hit the workpiece. For this purpose, suitable Python or Matlab scripts are used [30,32,37,38].

In addition to the above, the shot-peening process can be used to improve the dimensional and shape accuracy of manufactured elements. In some situations, the use of shot peening makes it possible to use construction materials that have lower mechanical properties or were not heat-treated; this is possible because these properties can be improved after the shot-peening process as a result of work hardening by shot peening.

Moreover, there are other innovative techniques, such as ultrasonic nanocrystal surface modification [39,40]. This method implements ultrasonic shocks in the designed path to induce surface hardening and reduce roughness. Similar effects are obtained after the shot-peening process.

Shot peening usually leads to changes in surface roughness, wear resistance, corrosion resistance, and fatigue strength, with the extent of these changes depending on the method and process conditions applied.

## 2. Motivation

The shot-peening process can be performed in many ways: centrifugal (which can produce significantly higher impact energy [41]), blasting, or vibratory shot peening.

**Figure 2:**
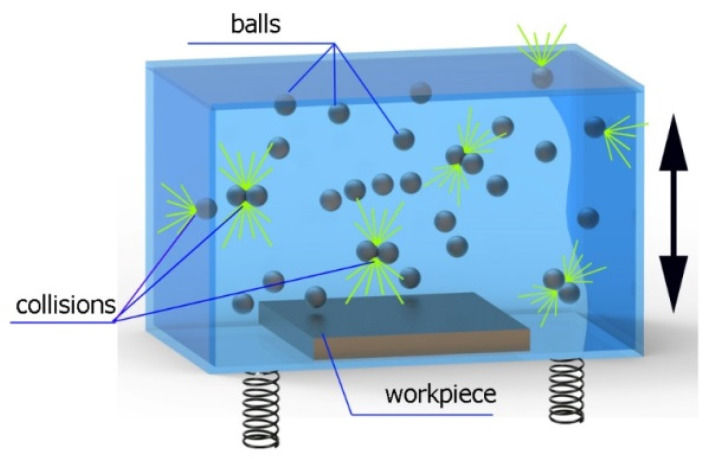
Schematic representation of the vibratory shot-peening process.

Figure 2 shows a schematic representation of the vibratory shot-peening process. A sample and balls are placed in a container that is made to vibrate with a specific amplitude. The sample is attached to the bottom of the container. Under these conditions, the shot-peening elements have different (unknown) speeds when they hit the machined surface; therefore, their impact energies differ.

As a result of the container’s vibration, the balls move chaotically when hitting the surface of the sample. Given the fact that the balls collide both with one another and with the walls of the container, which—consequently—changes the direction of their movement and impact energy, it is impossible to determine the distribution of the location of dimples on the machined surface. Visualizations of the effects of this process and the phases of dimple formation in vibratory peening are given in Table 1. For better visualization, the process of applying the 36 dimples was divided into 4 phases (one after the other). The distribution of dimples is random, and the size of cavities made by the peening elements varies due to the loss of energy that occurs when the balls collide either with one another or against the walls of the container. The process is usually continued until the surface is completely covered with the dimples formed due to the impact of the peening elements. Since a mathematical description of this process type is very complex, when analyzing the influence of input factors on treatment effects, only intermediate parameters, i.e., the amplitude and frequency of device vibration, are given instead of impact speed and energy.

Blasting is similar to vibratory shot peening. This process is also difficult to describe and model mathematically. In blasting, the surface is hit by high-speed moving balls (usually set in motion by compressed air).

**Figure 3:**
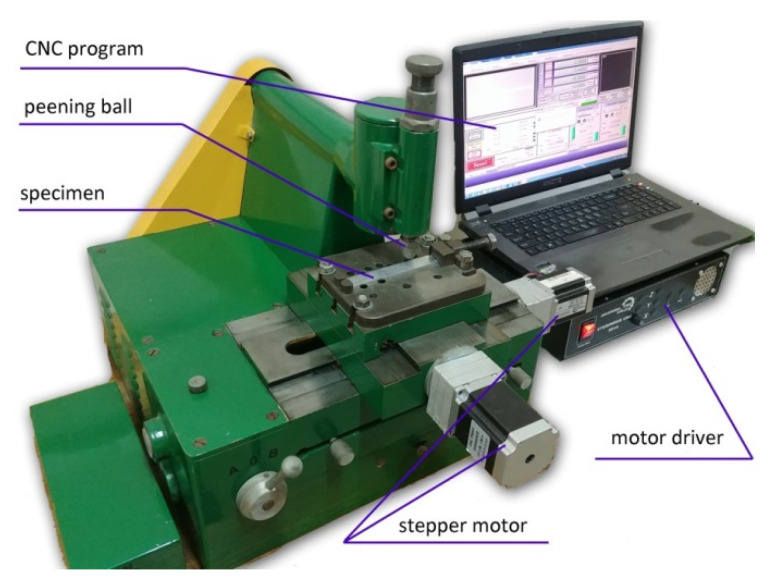
View of the RSP and SRSP test stand.

The semi-random-shot-peening (SRSP) method is an alternative to vibratory shot peening and shot blasting. This method makes it possible to control impact energy, as well as the distribution and sequence of dimples. Figure 3 shows the test stand developed by the authors for SRSP and regular shot-peening (RSP) testing. The device is equipped with exchangeable heads that enable changing the diameter of the ball-shaped peening element. The use of a cam element and spring allows the impact energy to be varied. The shot-peened sample is mounted on the CNC table, which moves according to the assumed dimpling schedule.

Table 2 shows individual phases of dimple creation in semi-random shot peening. The phases of the process are shown in the form of surface topographies and real photographs of individual dimples. In the initial phases of the process, the distance between individual dimples is greater than the diameters of the dimples (there is no “contact” between the dimples). In the final phase of the process, the dimples uniformly cover the machined surface, which provides greater uniformity in comparison with vibratory shot peening.

In the conventional impulse peening process, when the peening element hits the surface, the dimples are formed in an orderly sequence with a regular distance between them. This process is known as regular shot peening (RSP). Individual phases of dimple formation are shown in Table 3.

**Figure 4:**
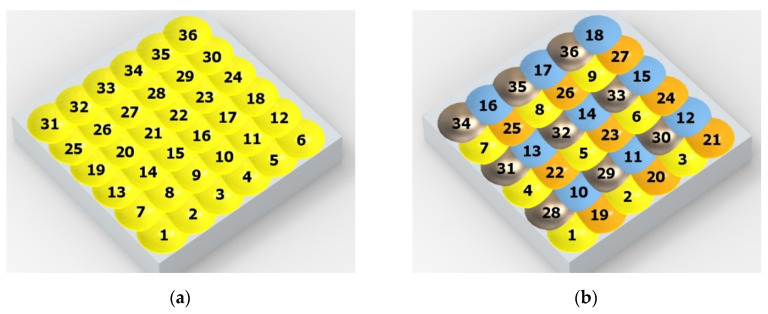
Comparison of the dimple formation methods: (**a**) RSP, (**b**) SRSP.

Figure 4 shows the visualization of dimples made with the two presented impulse-peening methods (RSP and SRSP). Impact intensity (the number of impacts per unit area) was the same for both methods (with the same distance maintained between individual dimples, as determined in the process). Nevertheless, the results obtained after RSP and SRSP differ with respect to surface roughness and microhardness distribution, as well as to plastic strain depth. This is caused by, among other things, the phenomenon of ridge formation after impact.

**Figure 5:**
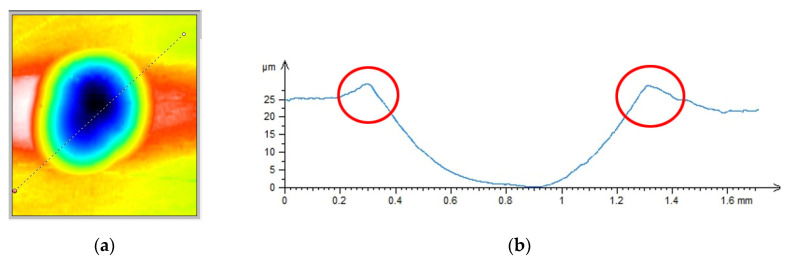
Dimple after impact: (**a**) dimple topography, (**b**) cross-section.

Figure 5 shows the dimple topography after a single impact (Figure 5a) and the cross-section to illustrate the size of a formed ridge (Figure 5b). The RSP method ensures that the distribution of material hardening (caused by a previous impact) is similar throughout the material (compared to SRSP).

As for the SRSP method, surface hardening caused by previous impacts differs depending on the impact phase (Table 2), which may result in a different degree of surface layer hardening. The dimples produced in the first phase are spaced apart from one another, and the ridge formed due to plastic deformation induced by the shot-peening element is symmetrical and evenly distributed around the dimple. In the subsequent phases of the process, the ratio of deformed to undeformed surface area differs, which affects surface roughness and its topography.

The aim of this study is to evaluate the effect of RSP and SRSP, as well as technological parameters of these shot-peening techniques (impact energy, ball diameter, and distance between dimples) on selected properties of the surface layer.

## 3. Materials and Methods

### 3.1. General Methodology

**Figure 6:**
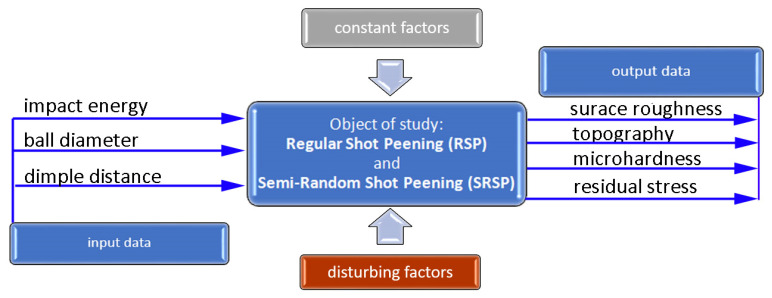
Diagram illustrating the methodology of the shot-peening process.

A general methodology of the study is delineated in Figure 6. The object of the study was two shot-peening techniques: Regular Shot Peening (RSP) and Semi-Random Shot Peening (SRSP). Different ball diameters, impact energies, and distances between dimples were used. The constant factors were: workpiece material, specimen shape, and test stand. Testing was conducted on the original test stand shown in Figure 3. The effect of input parameters on surface roughness, topography, and microhardness was investigated. A FEM simulation was performed to determine residual stresses and their depth distribution.

Surface roughness and topography were obtained from the T8000RC120-400 profilographometer provided by Hommel–Etamic Jenooptik (Jena, Villingen-Schwenningen, Germany). Surface microhardness was analyzed with the Leco LM700 device in compliance with the EN-ISO 6507-1:2018 standard. A 50 g load was applied; the penetrator loading time was 15 s.

### 3.2. Materials

Specimens of EN AW 7075 aluminum alloy were used in the experiment. Table 4 presents the chemical composition and physical properties of the tested material.

Due to its properties, this alloy is widely used in the aviation and automotive industries. Cuboid samples of the tested material, each having the dimensions of 100 × 15 × 4 mm, were used for testing.

### 3.3. Shot-Peening Parameters

Tests were performed using two methods: regular shot peening (RSP) and semi-random shot peening (SRSP). The two dimple formation methods are described in Section 2 and shown in Table 2 and Table 3. Individual phases illustrate the shot-peening process that was carried out for the entire surface. In the experiment, impact energy, ball diameter, and distances between dimples were analyzed. Table 5 lists the applied shot-peening parameters.

### 3.4. Numerical Simulations of the Shot-Peening Process

**Figure 7:**
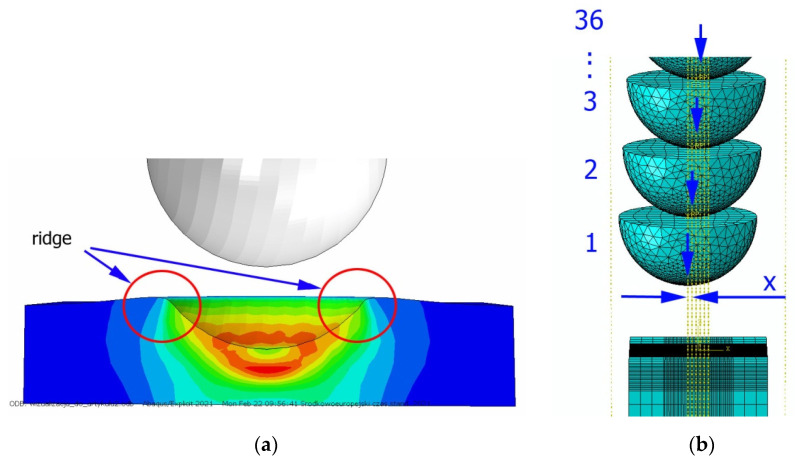
FEM simulation of the shot-peening process: (**a**) view of a single dimple with a ridge, (**b**) visualization of 36 impacts according to the applied methodology.

A numerical analysis of the shot-peening process was performed in the Explicit module of the Abaqus CAE software, considering surface-to-surface contact. The Johnson–Cook constitutive model was used with the following parameter A = 503 MPa, B = 678 MPa, n = 0.71, C = 0.024, m = 1; the model considers the effect of strain hardening, strain rate, and temperature on the stress−strain relation. Impact energy was introduced by taking into account the mass and speed of the peening element. Numerical results (dimple diameters) were then compared with experimental findings. For the numerical model of a 10 × 10 × 4 mm specimen, elements of type C3D8R were used. The element size of contact area was reduced to 0.1 mm. The total number of elements in the mesh was 68,992, with the amount equal to 74,727 nodes. The ball-shaped peening element was modeled as a rigid body using two types of elements: R3D4 (476 elements) and R3D3 (2616 elements). The mesh size was reduced in the workpiece contact area. The S11 stress state corresponding to the residual stresses in the surface layer after the burnishing process and the PEEQ equivalent plastic strain were analyzed. Figure 7a shows an example visualization of a dimple together with a visible ridge on the cross-section.

**Figure 8:**
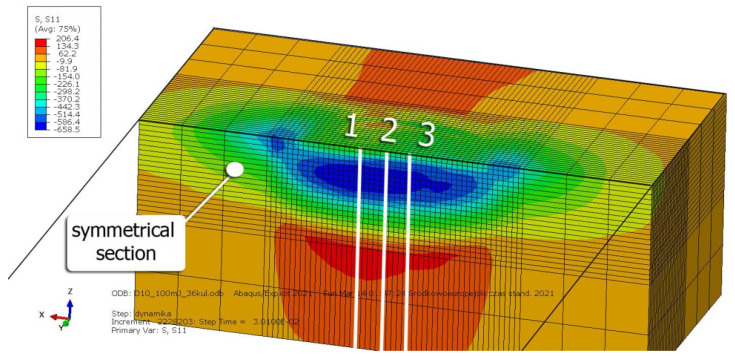
Method of determination the averaged S11 stress distribution based on three paths.

Figure 7b shows a visualization of 36 impacts with an *x* distance between the dimples. To evaluate the influence of individual parameters, FEM simulations of 36 impacts were performed for the parameters listed in Table 5 for both SRSP (according to the methodology described in Table 2) and RSP (according to the methodology described in Table 3). S11 stress plots from the FEM simulations were determined as the average value from three cross-section paths drawn perpendicular to the surface, as shown in Figure 8.

## 4. Results

### 4.1. Surface Roughness and Topography after SRSP and RSP

**Figure 9:**
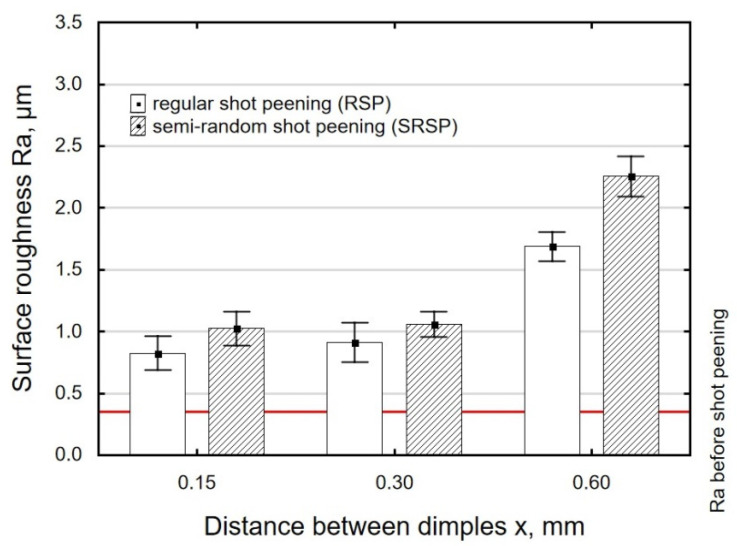
Distance between dimples versus roughness parameter Ra (impact energy E = 100 mJ, ball diameter D = 10 mm).

Figure 9 shows the effect of the distance between the dimples on the Ra roughness parameter. It can be observed that increasing the distance from 0.15 to 0.3 did not lead to any considerable increase in the roughness parameter; however, when the distance was increased from 0.3 to 0.6, it caused a significant increase in the Ra parameter. The use of larger distances between the dimples reduced the degree of dimple coverage.

**Figure 10:**
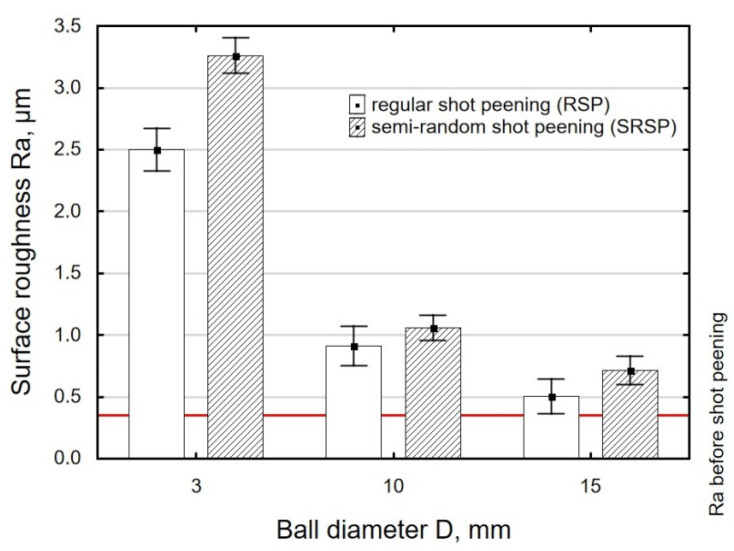
Shot-peening element diameter versus roughness parameter Ra (impact energy E = 100mJ, distance between dimples x = 0.3 mm).

On the other hand, an increase in the diameter of the peening element led to reduced roughness (Figure 10). For the peening element with a diameter of 3 mm, clearly higher roughness values can be observed when compared to the elements with diameters of 10 and 15 mm. This results from the fact that the contact area for the 3 mm diameter peening element is smaller, and thus the pressures increase (with the impact energy maintained constant), which produces greater surface irregularities.

**Figure 11:**
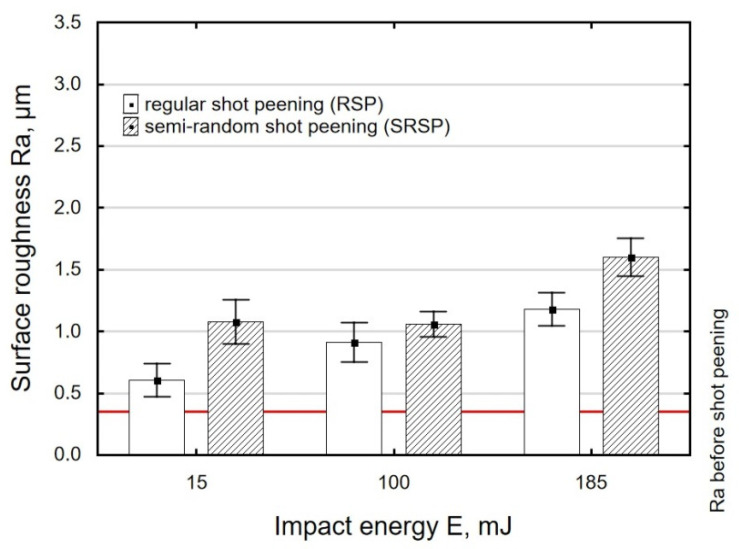
Impact energy versus roughness parameter Ra (ball diameter D = 10 mm, distance between dimples x = 0.3 mm).

With an increase in the impact energy, there is a slight increase in surface roughness, as shown in Figure 11. It should be noted that in all cases under study, the achieved roughness values are higher than the initial roughness (horizontal line in Figure 8, Figure 9 and Figure 10) obtained after grinding.

For the tested parameter ranges, the Ra parameter values are higher after SRSP than after RSP.

Table 6, Table 7 and Table 8 show the effects of the distance between the dimples, ball diameter, and impact energy on the Sa roughness parameter for RSP and SRSP, respectively. For the entire range of the analyzed input factors, the values of the Sa parameter are higher for the SRSP process. It can be observed that surface roughness increases with increasing the distance between the dimples (Table 6).

The lowest values of the Sa parameter can be observed for the peening element with the largest diameter (with the distance between the dimples maintained constant, Table 7). On the other hand, an increase in the impact energy leads to a greater deformation of the workpiece surface (Table 8).

### 4.2. Microhardness after SRSP and RSP

**Figure 12:**
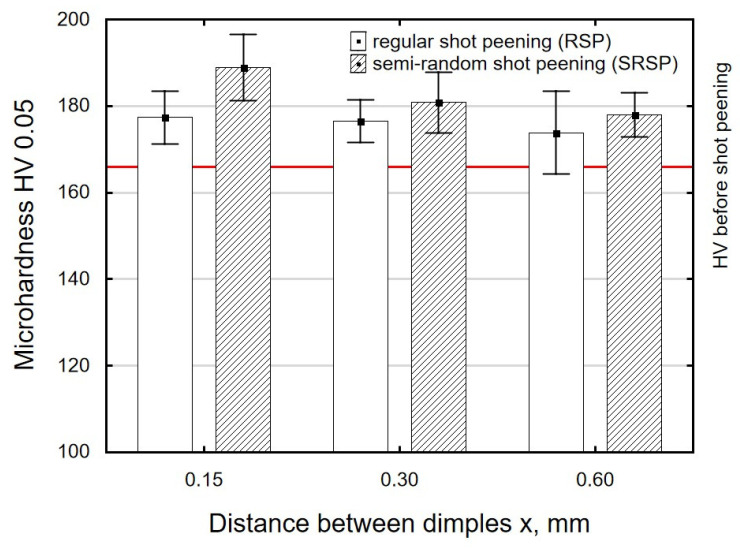
Distance between the dimples versus surface microhardness (impact energy E = 100 mJ, ball diameter D = 10 mm).

**Figure 13:**
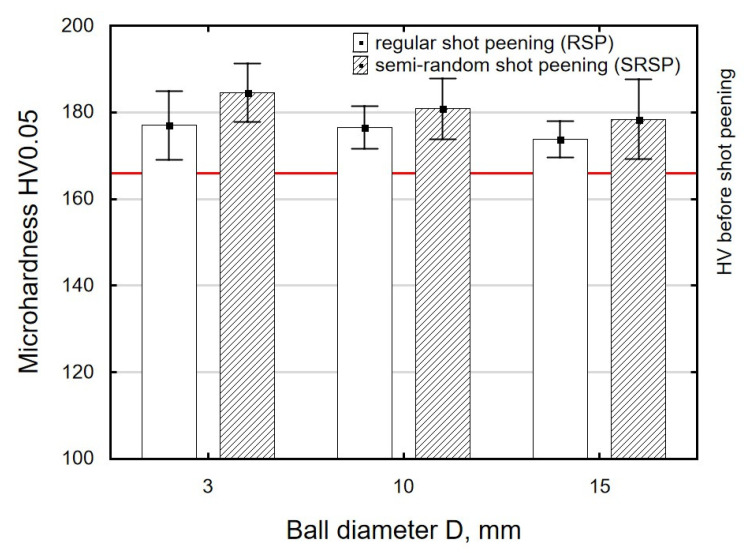
Peening element diameter versus surface microhardness (impact energy E = 100 mJ, distance between dimples x = 0.3 mm).

**Figure 14:**
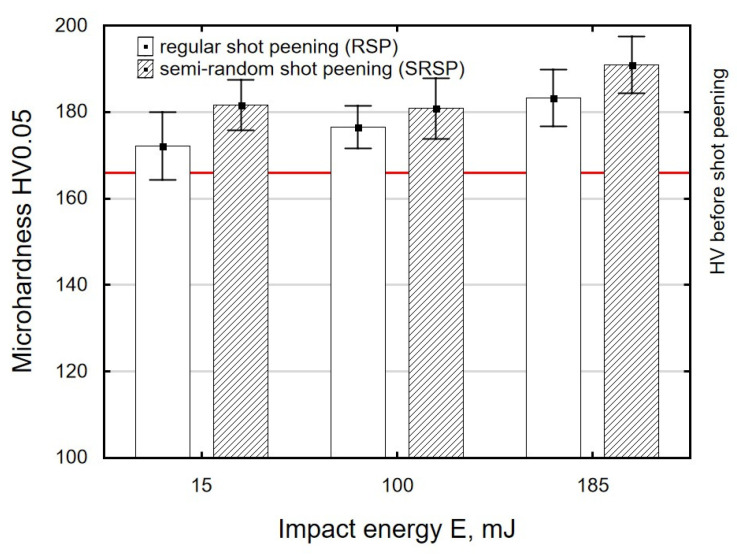
Impact energy versus surface microhardness (ball diameter D = 10 mm, distance between dimples x = 0.3 mm).

Figure 12, Figure 13 and Figure 14 show the effects of the distance between the dimples, ball diameter, and impact energy on the surface layer microhardness for RSP and SRSP, respectively. The horizontal line marks the microhardness value before the shot-peening process. A decrease in the distance between impacts leads to increased microhardness (Figure 12). On the other hand, when the diameter of the peening element is smaller, microhardness increases (Figure 13). This is caused by increased pressures due to a smaller contact surface between the peening element and the workpiece. With increasing the impact energy, the microhardness of the surface increases, too (Figure 14).

For the entire range of the analyzed input factors, a higher value of microhardness can be observed for the SRSP method than for the RSP method. This is caused, among other things, by hitting the ridge that was formed as a result of an earlier impact. The surface layer of the ridge area may be characterized by a greater degree of hardening.

In addition, greater surface irregularities (ridge shape) may lead to a reduction in the real contact area between the burnishing element and the workpiece and, consequently, to higher stresses, which—in turn—leads to increased microhardness.

### 4.3. FEM Simulation of Shot Peening

#### 4.3.1. Comparison of Real and FEM Dimple Diameters

**Figure 15:**
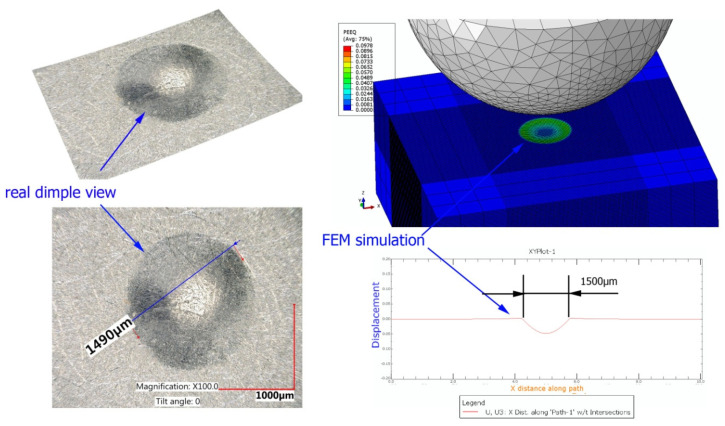
Comparative visualization of a single dimple (impact energy E = 100 mJ, ball diameter D = 10 mm).

To assess the correctness of the numerical simulations, a series of single dimples were made experimentally for the applied experimental conditions (impact energy and peening element diameter). For comparative purposes, a FEM numerical simulation was performed for the same conditions, as illustrated by the example in Figure 15.

**Figure 16:**
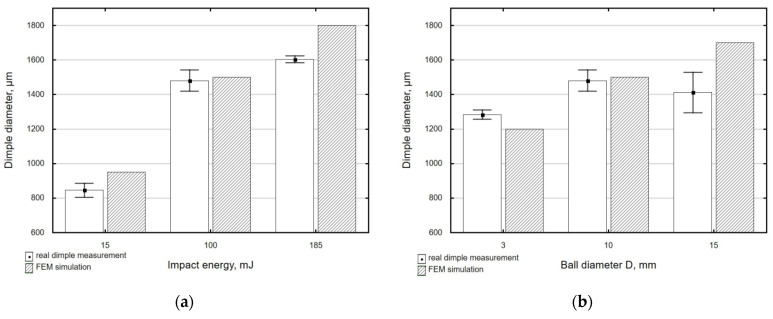
Comparison of the real and FEM numerical simulation dimple diameters as a function of: (**a**) impact energy, (**b**) shot-peening element diameter.

Comparative results of the real dimples and those obtained by FEM numerical simulation for the selected peening element diameters and impact energies are given in Figure 16.

The greatest difference between the experimental and FEM results of dimple diameters can be observed for a ball diameter of D = 15 mm. For this largest tested diameter of the peening element, the measurement error is the highest due to the smallest ratio of dimple depth to diameter, which is confirmed by the highest values of the standard deviation for the dimples obtained for the 15 mm diameter ball.

#### 4.3.2. FEM Simulation of PEEQ Equivalent Plastic Strain and S11 Stress Distributions

Table 9, Table 10 and Table 11 show the visualization of PEEQ equivalent plastic strain after 36 impacts of the peening element, respectively, for RSP and SRS. PEEQ is a scalar variable that is used to represent the material’s inelastic deformation and to determine the percentage (after multiplication ×100) of plastic strain in relation to the initial state (in the analyzed area). To visualize strains on the surface as well as in the subsurface layers, a symmetrical cross-section was made. The color maps below of PEEQ equivalent plastic strains reveal that the largest plastic strains are located at some distance from the surface.

With decreasing the diameter of the peening element, the stresses in the surface layer increase, which—in turn—leads to an increase in the PEEQ value. An increase in the impact energy leads to an increase in the equivalent plastic strain. For all analyzed cases, higher PEEQ values were obtained after SRSP than after RSP.

**Figure 17:**
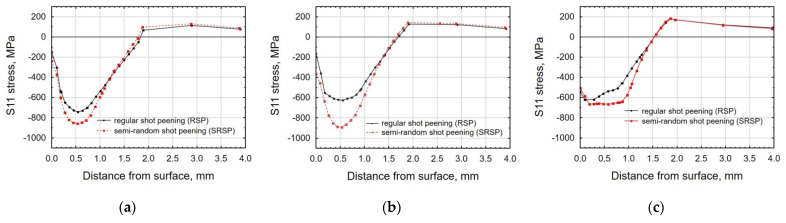
Comparison of the S11 stress distribution for RSP and SRSP as a function of distance from the surface for different distances between the dimples: (**a**) x = 0.15 mm, (**b**) x = 0.3 mm, (**c**) x = 0.6 mm.

**Figure 18:**
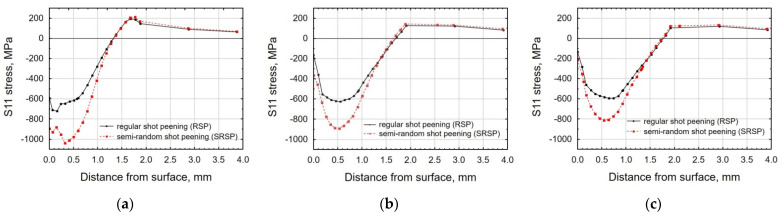
Comparison of the S11 stress distribution for RSP and SRSP as a function of distance from the surface for different ball diameters: (**a**) D = 3 mm, (**b**) D = 10 mm, (**c**) D = 15 mm.

**Figure 19:**
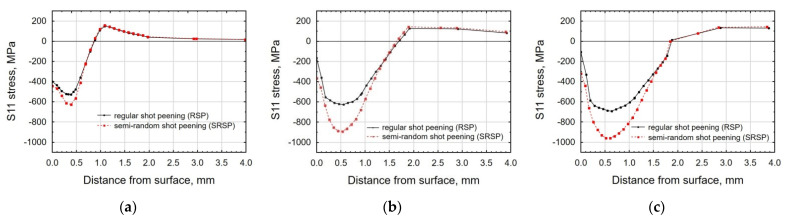
Comparison of the S11 stress distribution for RSP and SRSP as a function of distance from the surface for different impact energies: (**a**) E = 15 mJ, (**b**) E = 100 mJ, (**c**) x = 185 mJ.

Figure 17, Figure 18 and Figure 19 show the effects of the distance between the dimples, ball diameter, and impact energy on the S11 stress distribution in the surface layer for RSP and SRSP, respectively. Higher S11 stress values can be observed for the SRSP method.

As shown in Figure 19, when the impact energy increases, both the maximum compressive stress and the depth of the stress increase.

## 5. Conclusions

In this paper, regular shot peening (RSP) and semi-random shot peening (SRSP) were compared. A characteristic of the first method is that the peening elements hit the treated surface in sequence and thus maintain a regular distance between the dimples formed. The other method (SRSP) is a controlled modification of the shot-peening process, which is random by nature. This study was conducted under variable process conditions. The following summarizes the results of the study investigating the influence of shot peening on the treatment effects:

- In the whole range of variability of the applied shot-peening parameters, higher values of the Ra parameter were obtained for SRSP than for RSP: from 16% (for E = 100 mJ, x = 0.3 mm, D = 10 mm) to 78% (for E = 15 mJ, x = 0.3 mm, D = 10 mm);
- The lowest value of the roughness parameter Ra, 0.5 µm, was obtained for RSP conducted using E = 100 mJ, x = 0.3 mm, D = 15 mm;
- Higher values of the Sa parameter were obtained for SRSP than for RSP—the highest difference (Sa = 1.88 µm for RSP and Sa = 4.47 µm for SRSP) was observed for the following parameters: E = 100 mJ, x = 0.6 mm, D = 10 mm;
- Higher roughness parameters were observed after RSP and SRSP alike when compared to the treatment before shot peening;
- Higher values of the surface layer microhardness were obtained after SRSP than after RSP;
- After RSP, the microhardness of the surface increased about ΔHV0.05 = 6 ÷ 17, while the surface microhardness increased;
- After, SRSP was ΔHV0.05 = 12 ÷ 25 compared to the surface prior to shot peening;
- Considering the entire range of parameter variation, the average microhardness of the surface increased by 6% after RSP and by 10.5% after SRSP compared to the surface microhardness before shot peening;
- Compressive residual stresses occur in the surface layer after the RSP and SRPS processes. The maximum compressive residual stresses were higher after SRPS than after RSP;
- Higher values of the PEEQ equivalent plastic strain were observed after SRSP than after RSP;
- The highest PEEQ value was obtained for SRSP conducted with a 3 mm peening element.

## Figures and Tables

**Table 1:** Methodology of exerting a random sequence of impacts in vibratory peening.

	Visualization of the Sequence of Dimples	Surface Topography	Real View of Surface
Phase 1	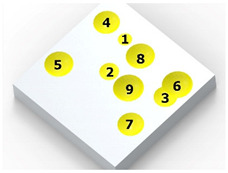	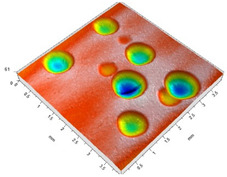	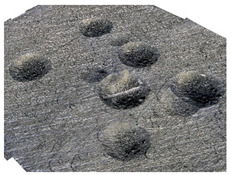
Phase 2	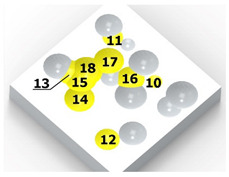	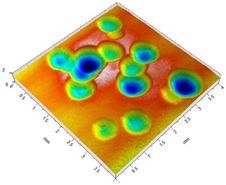	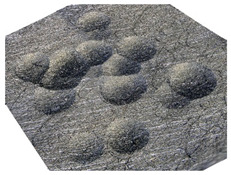
Phase 3	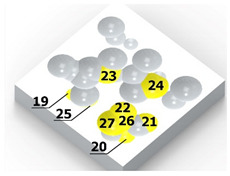	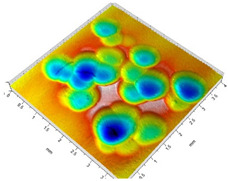	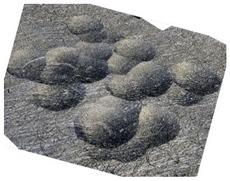
Phase 4	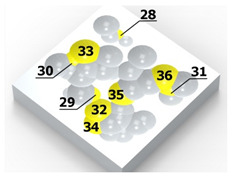	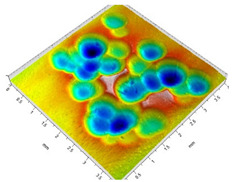	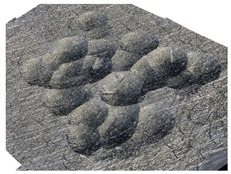

**Table 2:** Methodology of exerting a sequence of impacts in semi-random shot peening (SRSP).

	Visualization of the Sequence of Dimples	Surface Topography	Real View of Surface
Phase 1	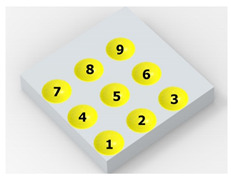	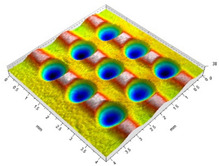	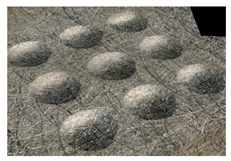
Phase 2	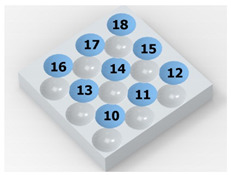	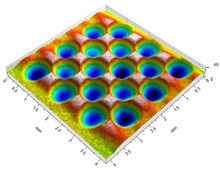	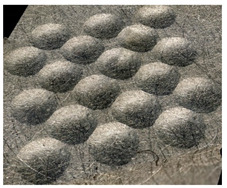
Phase 3	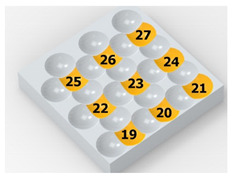	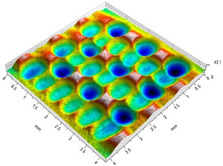	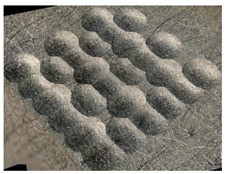
Phase 4	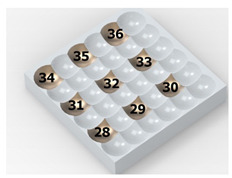	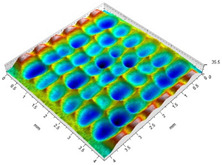	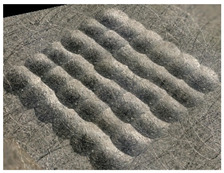

**Table 3:** Methodology of exerting a sequence of impacts in regular shot peening (RSP).

	Visualization of the Sequence of Dimples	Surface Topography	Real View of Surface
Phase 1	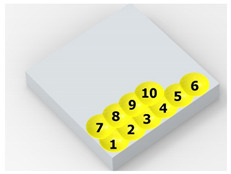	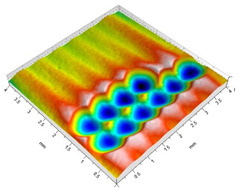	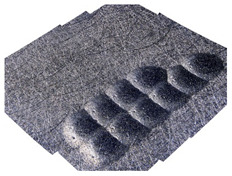
Phase 2	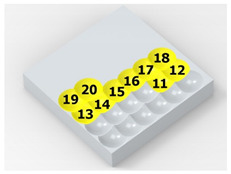	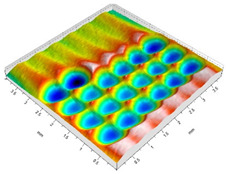	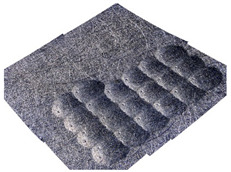
Phase 3	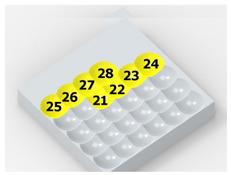	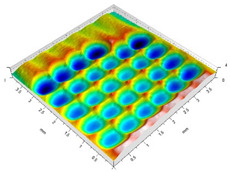	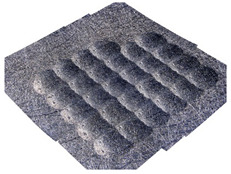
Phase 4	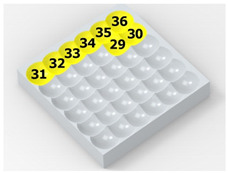	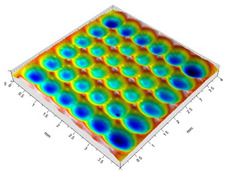	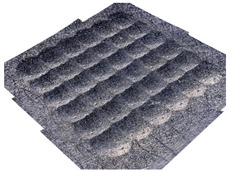

**Table 4:** Chemical composition and physical properties of EN AW 7075 aluminum alloy.

Chemical Composition, wt.%		Physical Properties	
Cu	1.59	Rm, MPa	572
Mn	0.01		
Mg	2.56		
Cr	0.18	Rp_0.2_, MPa	503
Zn	5.78		
Si	0.07		
Fe	0.13	HB	166
Ti	0.05		
Al	Rest		

**Table 5:** Shot-peening conditions.

No.	Impact Energy E (mJ)	Ball Diameter D (mm)	Distance between Dimples x (mm)
1	100	10	0.3
2	100	10	0.15
3	100	10	0.6
4	100	3	0.3
5	100	15	0.3
6	15	10	0.3
7	185	10	0.3

**Table 6:** Effect of the distance between the dimples on the Sa roughness parameter and surface topography (impact energy E = 100 mJ, ball diameter D = 10 mm).

	Distance between Dimples x (mm)		
	0.15	0.3	0.6
RSP	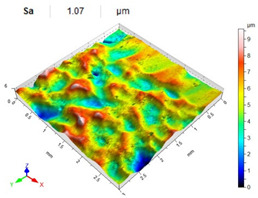	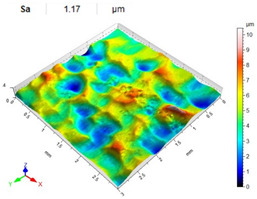	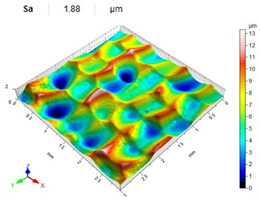
SRSP	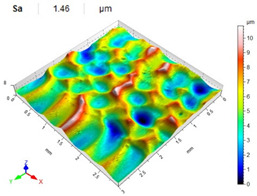	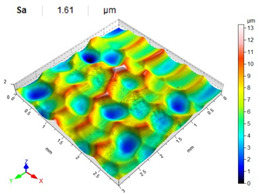	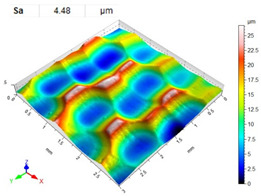

**Table 7:** Effect of the shot-peening element diameter on the Sa roughness parameter and surface topography (impact energy E = 100 mJ, distance between dimples x = 0.3 mm).

	Ball Diameter (mm)		
	3	10	15
RSP	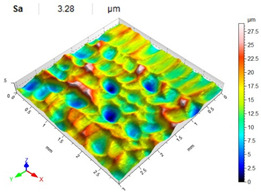	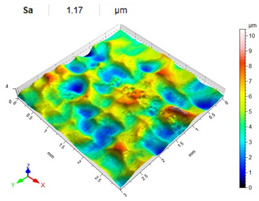	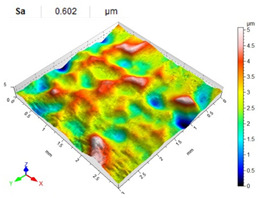
SRSP	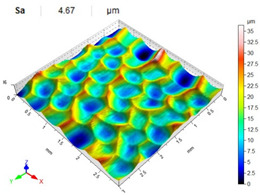	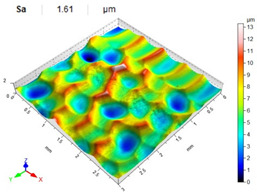	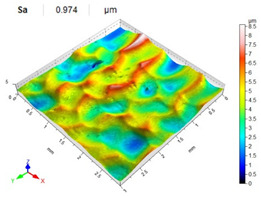

**Table 8:** Effect of impact energy on the Sa roughness parameter and surface topography (ball diameter D = 10 mm, distance between dimples x = 0.3 mm).

	Impact Energy E (mJ)		
	15	100	185
RSP	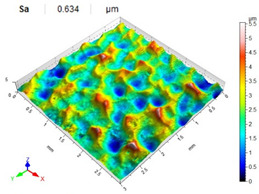	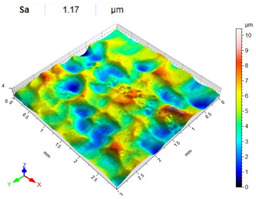	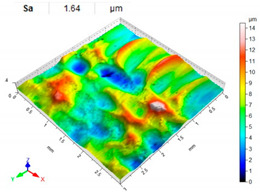
SRSP	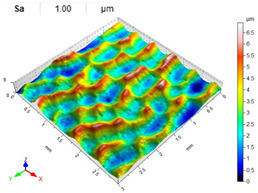	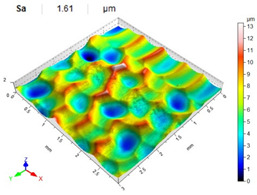	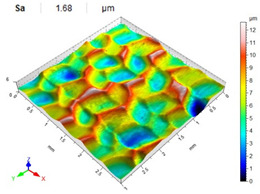

**Table 9:** Effect of the distance between the dimples on the value of PEEQ equivalent plastic strain after 36 impacts (impact energy E = 100 mJ, ball diameter D = 10 mm).

	Distance between Dimples x (mm)		
	0.15	0.3	0.6
RSP	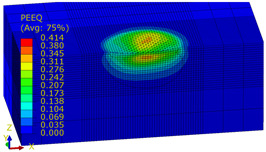	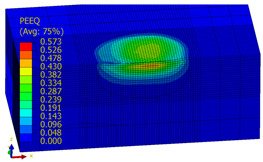	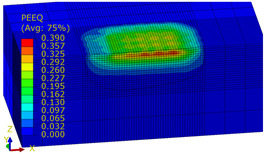
SRSP	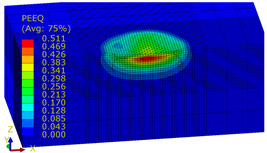	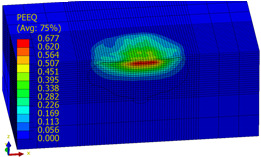	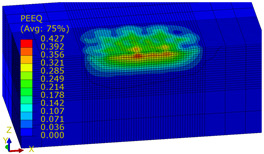

**Table 10:** Effect of the peening element diameter on the PEEQ equivalent plastic strain after 36 impacts (impact energy E = 100 mJ, distance between dimples x = 0.3 mm).

	Ball Diameter (mm)		
	3	10	15
RSP	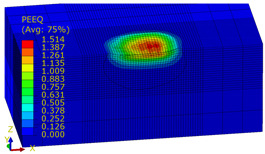	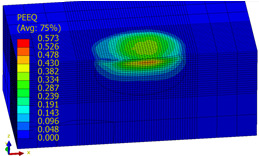	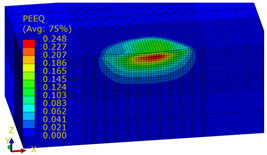
SRSP	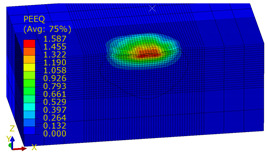	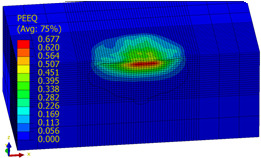	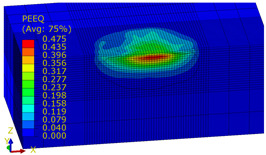

**Table 11:** Relationship between impact energy and PEEQ equivalent plastic strain after 36 impacts (ball diameter D = 10 mm, distance between dimples x = 0.3 mm).

	Energy (mJ)		
	15	100	185
RSP	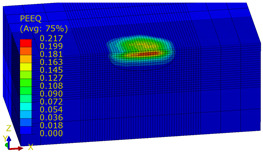	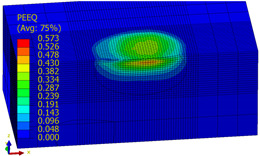	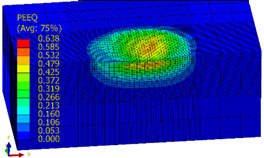
SRSP	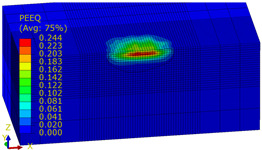	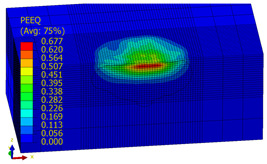	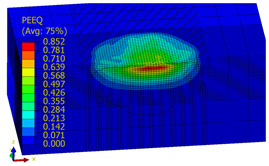

## Data Availability

The data presented in this study are available on request from the corresponding author.
